# On the Possibility of Supporting Life on a Vegetable Diet

**Published:** 1845-01

**Authors:** Wm. Lambe

**Affiliations:** Bromptly


*On the Possibility of Supporting Life on a Vegetable Diet.*

To the Editor of the Lancet:

Sir: In the *Times* newspaper, of the 4th inst., you are reported to have said, "that you had found, to your surprise, that for the first fortnight of an untried prisoner's incarceration he was subjected to the lowest possible diet, being denied, in fact, all animal food for the first fourteen days of his confinement;" you obviously would represent this restriction from animal food as a most inhuman and injurious treatment.

I apprehend it to be impossible for you not to know that the experience of all ages has proved that the healthy man can be perfectly nourished without using a particle of animal food. I will fearlessly assert, from long experience, that vegetable food is much more salubrious than mixed diet in common use, in which, however, animal matter commonly enters in the smallest proportion. Numerous instances may be cited of persons who have lived for years in very good health without animal food; but I will here content myself with very shortly informing you of my personal experience in this matter.

In the year 1804 (in January) I resolved to confine myself to a strict vegetable diet; I was induced to this by severe bodily suffering. In this course I have persevered, without deviation, to the present time, now more than thirty-eight years, and by its means have now advanced in my eightieth year. The diet has been aided by other measures, which I need not enter into. I have brought up a large family on the same plan with perfect success. My eldest son, who was in childhood very delicate, with an obvious consumptive tendency, has used the same regimen for an equal length of time, and is in perfect health; and I could cite numerous instances of its beneficial effects in various individuals.

I am no advocate for starvation, and opposed, certainly, to a mere bread-and-water diet. Indeed, I cannot approve of any fixed dietary, it being abhorrent to nature, which delights in variety; the vegetable kingdom affords variety sufficient to satisfy any healthy appetite.

I have addressed to you these facts, as in the station you hold as a member of Parliament what falls from you on medical subjects may have considerable influence either for good or for evil, and I shall be sorry to see talents of no mean order employed to fortify vulgar prejudices, or to disseminate erroneous opinions. I am, sir, your obedient servant,

WM. LAMBE, M.D.

Bromptly, June 6, 1844.

